# Papillary tumor of the pineal region: A case report and review of the literature

**DOI:** 10.3892/etm.2015.2696

**Published:** 2015-08-20

**Authors:** XIAOMIN HUA, PING YANG, MING ZHANG, YUDAN ZHAO, BIN WANG

**Affiliations:** 1 Department of Microbiology, Qingdao University Medical College, Qingdao, Shandong 266071, P.R. China; 2 Department of Pathology, Yantai Yuhuangding Hospital, Yantai, Shandong 264000, P.R. China; 3 Department of Anatomy, Qingdao University Medical College, Qingdao, Shandong 266071, P.R. China

**Keywords:** papillary tumor of the pineal region, pineal region, immunohistochemistry, central nervous system, differential diagnosis

## Abstract

Papillary tumor of the pineal region (PTPR) was first described as a distinct tumor entity in 2003 and was introduced into the World Health Organization classification of central nervous system tumors in 2007. This tumor is rare and, to the best of our knowledge, only 7 cases have been reported in children <16 years of age, while the youngest documented patient was a 15-month-old boy. The present study reported a case of PTPR in a 10-year-old girl who underwent magnetic resonance imaging and surgical resection of tumors. Histological and immunohistochemical staining results were presented. Patients with PTPR require long-term follow-up, and the patient of the present study has continued to do well, with no recurrence of the tumor at the 15-month follow-up examination. In addition, a review of the literature on this unusual neoplasm was performed, along with discussion of their differential diagnosis.

## Introduction

Papillary tumor of the pineal region (PTPR) was originally described as a distinct clinicopathological entity by Jouvet *et al* in 2003 (1). In 2007, PTPR was included in the World Health Organization classification of central nervous system tumors (2). PTPR does not arise from the pineal gland itself, but originates from specialized cytokeratin-positive and nestin-positive ependymal cells that are derived from the subcommissural organ (3–5). Tumors of the pineal region are rare lesions, accounting for only 1% of all intracranial tumors (6). PTPR have morphological features in common with a number of other papillary-like tumors that occur in the pineal region, including pineal parenchymal neoplasms, choroid plexus papilloma, papillary ependymoma, metastatic papillary carcinomas, papillary meningioma and germ cell tumors (5,7), which complicates the clinical diagnosis of PTPR. Clinical presentation most often includes headache and obstructive hydrocephalus. Microscopic evaluation often demonstrates a lesion with papillary areas lined by epithelioid tumors with eosinophilic cytoplasm, and numerous cells exhibiting clear or vacuolated cytoplasm. Perivascular and true rosettes may be identified (8). The natural history and optimal treatment of PTPR remain controversial (9) and Kaplan-Meier analysis provided a 5 year survival estimate of 73% (10). The present study reports the case of a 10-year-old patient that underwent magnetic resonance imaging (MRI) and surgical resection of tumors of the pineal region. The final diagnosis of PTPR was based on the morphological features of the tumor cells and the results of immunohistochemical staining. A written informed consent was obtained from the patient's family.

## Case report

**Figure 1.**
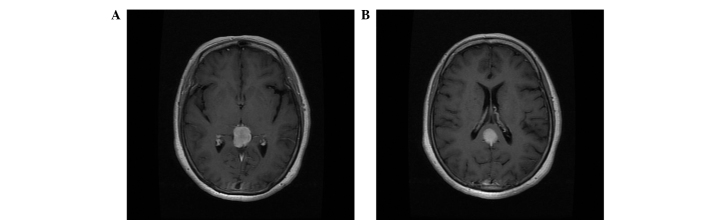
Axial magnetic resonance images demonstrating a well-circumscribed space-occupying lesion in the pineal region. (A) T1-weighted axial MRI of a well-circumscribed solid pineal tumor. (B) T1-weigthed axial MRI of a tumor and the ministry of corpus collosum. MRI, magnetic resonance imaging.

A 10-year-old girl presented with one-year history of right eye strabismus accompanied by diplopia, with no apparent cause. The patient was treated with traditional Chinese medicine in a local hospital and the diplopia symptoms were alleviated, while the strabismus symptoms persisted. One month prior to presentation, the patient suffered from an irregular intermittent headache, particularly in the lateral and top areas of the forehead. During this period, the patient additionally experienced intermittent nausea and vomiting. For further evaluation, the patient was admitted to the Affiliated Hospital of Qingdao University (Qingdao, China) in March 2014. The results of a physical examination conducted at the point of patient admission to the hospital were unremarkable with the exception of the right eye strabismus. Further MRI scans demonstrated a heterogeneously-enhanced and well-defined space-occupying lesion with limited cystic components in the pineal region (Fig. 1). The patient was diagnosed with hydrocephalus and abnormal cerebral aqueduct, which was considered to be a tumor.

The tumor was removed via a suboccipital transtentorial approach (11). During surgery, the tumor appeared grayish, soft, well-circumscribed and markedly vascular, exhibiting adhesion to the deep venous system and strong adhesion to the corpora quadrigemina. The majority of the tumor was succesfully removed, and the patient underwent an endoscopic third ventriculostomy for hydrocephalus management.

**Figure 2.**
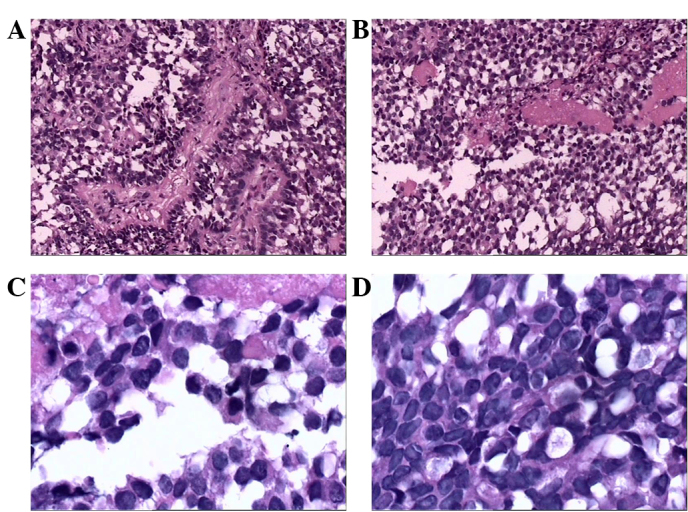
Hematoxylin and eosin staining revealing the papillary features of the tumor cells. (A and B) Sections of the tumor exhibited papillary structures and a palisade arrangement surrounding the vascular pseudo-stratified columnar epithelium (magnification, x100). (C and D) The cytoplasm was hyperchromatic and exhibited irregular nuclei (magnification, x400).

**Figure 3.**
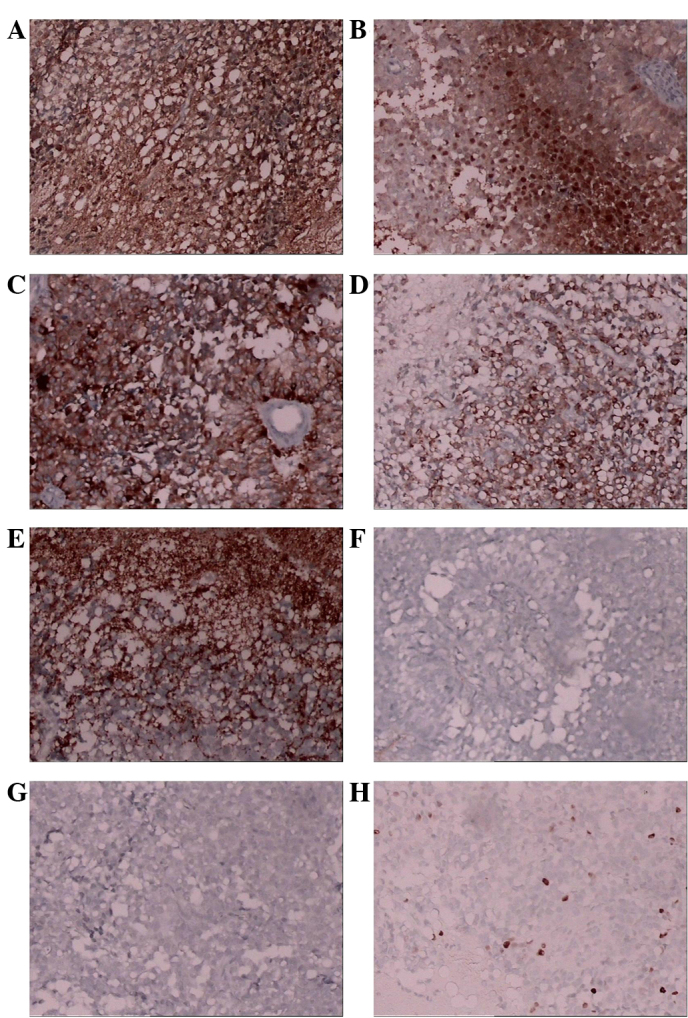
Micrographs demonstrating positive immunohistochemical staining for (A) S-100, (B) neuron-specific enolase, (C) Cam5.2, (D) CK8-18 and (E) Syn, and negative staining for (F) glial fibrillary acidic protein and (G) epithelial membrane antigen. (H) Approximately 5% of the tumor cells were positive for Ki67.

Microscopic examination revealed that parts of the tumor exhibited papillary structures and a palisade arrangement surrounding the vascular pseudostratified columnar epithelium was observed. Examination of hematoxylin and eosin-stained sections showed that the cells demonstrated papillary growth patterns. The cytoplasm was hyperchromatic and the nuclei were slightly irregular (Fig. 2). In addition, immunohistochemical staining revealed marked immunoreactivity for S100-protein, neuronal specific enolase, CAM5.2 and cytokeratin 8/18, while the tumor was focally immunoreactive for synaptophysin; however, the tumor was found to be negative for glial fibrillary acidic protein (GFAP) and epithelial membrane antigen. The Ki67 proliferative index at this initial resection was ~5% (Fig. 3). On the basis of these features, a diagnosis of PTPR was rendered. Postoperatively, the patient continues to do well, and no recurrent tumor was found at the 15-month follow-up examination.

## Discussion

The term ‘PTPR’ is based on the histopathological description of a tumor characterized by a papillary pattern, rosettes and pseudorosettes (11). Other tumors of the pineal region manifested by papillary features include pineal parenchymal neoplasms, choroid plexus papilloma, papillary ependymoma, metastatic papillary carcinomas, papillary meningioma and germ cell tumors. However, pineal parenchymal tumors, meningiomas and germ cell tumors rarely display papillary features (12). The immunohistochemical characteristics of PTPR include variable immunoreactivity for cytokeratin and S-100 protein, and complete absence of immunoreactivity for GFAP; therefore, GFAP staining aid in distinguishing this neoplasm from an ependymoma (13,14). Choroid plexus papilloma rarely occurred in the posterior third ventricle. Therefore, in the present study, ultrastructural and immunohistochemical analyses were used to distinguish this type of papillary tumor from other papillary-like tumors that occur in the region, and a final histological diagnosis of PTPR was confirmed.

PTPR is an uncommon type of neoplasm and, to the best of our knowledge, only 93 cases have been reported thus far (14–25). Table I summarizes 93 cases of patients with PTPR (14–25), including 74 cases described by Poulgrain *et al* in 2011 (15) and 19 cases reported after 2011 (8–25). The 93 previously reported cases include a wide range of ages. The youngest patient was a 15-month-old boy (16) and the oldest was a 67-year-old female (15). In addition, 7 cases have been reported in children younger than 16 years (Table II). The present study reported a case of PTPR in a 10-year-old girl, with the proportion of children being 7.53%. Notably, the incidence rates of PTPR in elderly patients are low and, to the best of our knowledge, only 2 cases have been reported in patients older than 65 years (15). By contrast, PTPR is more common among individuals aged ~30 years (7). Almost no difference was detected in PTPR prevalence between males and females in the cases described (47 males vs. 46 females). Tumors ranged between 5 and 49 mm in size. The recurrence rate was high (67.39%) and Kaplan-Meier analysis provided a 5-year survival estimate of 73% (10). In the present study we report a rare case of PTPR in a 10- year old girl who underwent a total tumour resection with no recurrence at the 15-month follow-up examination.

Clinical data concerning PTPR is limited and its pathogenesis is unknown. PTPR is frequently misdiagnosed as ependymoma or choroids plexus papilloma. However, the diagnostic criteria of certain postulated papillary-like tumors have been revised, and a more complete understanding of this tumor may be obtained. The present study reported a case of PTPR in a 10 year-old girl who suffered from an irregular intermittent headache, particularly in the lateral and top areas of the forehead. The patient were treated with Chinese medicine which alleviated the diplopia symptoms. Similarly to previous reports they underwent magnetic resonance imaging and surgical tumor resection, and continues to have a positive postoperative outcome. These results along with the data from previous studies indicate that total tumor resection is the optimal treatment guideline.

## Figures and Tables

**Table I.** Reports of patients with papillary tumor of the pineal region.

Year (Ref)	Cases (n)	Age (years)^a^/gender	Tumor size (mm)	Clinical symptoms	MRI features	Surgery type	Adjuvant therapy	Follow-up (months)	Recurrence/time post-surgery
2011 (12)	74	11–67 (M, 35; F, 39)	20–45	H/A, N, V memory loss	Well-circumscribed T1-hyperintensity CE	28 GTR 16 STR 30 NOS	36 RT 16 CT 20 ND, 2 Nil	0.75–218	12 months
2011 (13)	2	48/M, 36/F	21, 18	H/A, memory loss, diplopia, ataxia gait	CE	2 GTR	CT, ND	14 18	No
2011 (14)	1	15 months/M	10	Gait disturbance	Well-circumscribed	ND	CT	ND	ND
2011 (15)	1	47/F	30	Ataxia	Well-circumscribed	GTR	ND	36	ND
2011 (16)	3	30/F, 28/F, 32/M	ND	H/A, V, visual disturbances	Well-circumscribed	NOS	NOS	18	1 case/18 months
2012 (17)	1	3/F	35	H/A, N, gait instability	Inhomogeneous CE	GTR	CT	36	Yes/36 months
2012 (18)	1	22/M	ND	Diplopia	NOS	STR	ND	24	Yes/24 months
2012 (19)	2	48/M, 35/M	34	Mechanical falls, blurry vision	Well-circumscribed heterogeneously enhanced cystic	ND	ND	ND	ND
2012 (15)	1	47/M	30	Ataxia	Well-circumscribed	NOS	CT	48	Yes/36 months
2012 (20)	1	23/M	ND	H/A, left facial numbness	T1-hyperintensity	ND	CT	25	No
2012 (21)	1	32/M	10	H/A, visual disturbances	CE		ND	ND	ND
2013 (17)	1	31/M	10	H/A, confusion	3 masses with edema	ND	ND	84	Yes/12 months
2014 (22)	2	37/M, 45/F	49, 22	H/A, hypoacusis tinnitus, visual loss, imbalance urinary incontinence, ataxia	CE	2 NOS	CT, ND	108, 17	No
2014 (11)	1	23/F	ND	ND	ND	GTR	ND	9	Yes/3 months
2015 (23)	1	17/M	ND	H/A	T1-hyperintensity	NOS	ND	6	No

a Age provided in years, unless otherwise stated. Ref, reference list number; MRI, magnetic resonance imaging; CE, contrast enhancement; CT, chemotherapy; F, female; M, male; GTR, gross total resection; H/A, headache; N, nausea; V, vomiting; ND, no details; NOS, surgery but details not otherwise specified; STR, subtotal resection.

**Table II.** Clinicopathological characteristics of patients with papillary tumor of the pineal region in the reviewed literature.

Characteristic	Total cases, n	Percentage of the cases, %
Age, years		
≤16	7	7.53
16–65	84	90.32
≥65	2	2.15
Gender		
Female	46	49.46
Male	47	50.54
Size, mm		
≤10	3	10.71
>10	25	89.29
Recurrence		
Yes	31	67.39
No	15	32.61

Recurrence and size were not provided for all cases due to insufficient data.
